# Emerging infectious diseases and outbreaks: implications for women’s reproductive health and rights in resource-poor settings

**DOI:** 10.1186/s12978-020-0899-y

**Published:** 2020-04-01

**Authors:** Vijay Kumar Chattu, Sanni Yaya

**Affiliations:** 1 Department of Medicine, University of Toronto, Toronto, Ontario Canada; 2 Occupational Medicine Clinic, St. Michael’s Hospital, Unity Health Toronto, Toronto, Ontario Canada; 3 Global Institute of Public Health, Thiruvananthapuram, Kerala India; 4 School of International Development and Global Studies, Faculty of Social Sciences, University of Ottawa, Ottawa, Ontario Canada; 5 The George Institute for Global Health, The University of Oxford, Oxford, UK

**Keywords:** Sexual and reproductive health and rights (SRHRs), Emerging and re-emerging infectious diseases (EIDs), Low- and middle-income countries (LMICs), Sustainable developmental goals (SDGs), Gender, Women, Covid-19, Ebola, Zika, SARS

## Abstract

This century is witnessing dramatic changes in the health needs of the world’s populations. The double burden of infectious and chronic diseases constitutes major causes of morbidity and mortality. Over the last two decades, there has been a rise in infectious diseases, including the severe acute respiratory syndrome virus (SARS), the H1N1 pandemic influenza, the Ebolavirus and the Covid-19 virus. These diseases have rapidly spread across the world and have reminded us of the unprecedented connectivity that defines our modern civilization. Though some countries have made substantial progress toward improving global surveillance for emerging infectious diseases (EIDs), the vast majority of Low-and Middle-income Countries (LMICs) with fragile health systems and various system-related bottlenecks remain vulnerable to outbreaks and, as such, experience dramatic social and economic consequences when they are reported. Lessons learned from past outbreaks suggest that gender inequalities are common across a range of health issues relating to Sexual and Reproductive Health and Rights (SRHR), with women being particularly disadvantaged, partially due to the burden placed on them. Though these countries are striving to improve their health systems and be more inclusive to this vulnerable group, the national/ global outbreaks have burdened the overall system and thus paralyzed normal services dedicated to the delivery of Sexual and Reproductive Health (SRH) services. In this paper, we discuss the global commitments to SRH, the impact of the EIDs on the LMICs, the failure in the delivery of SRH services, and the strategies for successful implementation of recovery plans that must address the specific and differentiated needs of women and girls in resource-poor settings.

## Background

On March 11, 2020, the World Health Organization (WHO) declared Covid-19 a pandemic. According to the WHO, as of March 18, 2020, there were over 207,860 cases of Covid-19 and 8657 deaths in 166 countries [1]. There is no doubt the outbreak represents a tremendous public health threat to the world. This highly emerging pathogenic infectious disease and the panic it has caused are a stark reminder of how deeply interconnected our modern world has become.

According to the WHO, an emerging infectious disease is defined as one that either has appeared within a population for the first time, or has existed previously, but is rapidly spreading in terms of the number of people getting infected or in terms of new geographical areas. These diseases, which include Zika, Nipah, and the Avian Influenza (H5N1), are mainly zoonotic in origin [2]. In contrast, re-emerging diseases are defined as “diseases that appear after they have been on a significant decline which can be because of breakdown in public health measures for diseases that were under control or due to appearance of new strains of organisms” [3].

While biological sex differences regarding vulnerability to Covid-19 remain largely unknown, available evidence and lessons emerging from past global outbreaks of infectious disease suggest that pandemics more often than not, exacerbate vulnerabilities that are already present prior to the outbreak. The 2014 Ebola outbreak revealed that gender issues and women’s sexual reproductive health and rights (SRHR) were conspicuously invisible in both the short- and long-term international responses to the outbreak. The lack of inclusion of these issues exacerbated health inequities and social injustices that women were already facing.

The Guttmacher–Lancet Commission on SRHR anticipated in 2018 that almost all 4.3 billion people worldwide who are of reproductive age will have inadequate reproductive health services over the course of their reproductive years. The report also emphasized a holistic view of SRHR and identified many underlying issues and populations that are often neglected by health systems and the negative impact of social norms and national laws and policies on benevolent efforts aimed at improving the situation [4].

Currently, the Covid-19 outbreak is straining health systems in resource-poor settings. The transfer of already limited resources to deal with the outbreak may reduce access to sexual and reproductive health (SRH) and increase maternal and childhood mortality rates. Strict new quarantine measures can also affect health workers and closed borders may disengage women from productive work to sustain livelihoods and lead to structural barriers to the delivery of care.

The objective of the paper is primarily to discuss the impact of the recent global epidemics of emerging and re-emerging infectious diseases, highlight the huge impact of these diseases on women, demonstrate the value a gender-based analysis adds to recovery efforts and to programming, and inform future international advice and responses.

### Major global epidemics of emerging/re-emerging infectious diseases (EIDs) in this century

This century has witnessed many epidemics, with a significant number of them happening in LMICs. Some crossed national borders and became a regional concern, while others spread further and developed into global epidemics and pandemics. The epidemics were mainly caused by the presence of EIDs in the LMICs’ fragile health systems, systems that were already overburdened, understaffed, and not delivering the essential and life-saving services for women (e.g. birth delivery and abortion services) (Table 1).

**Table 1:** Summary of the recent global epidemics and their impacts on the health systems of LMICs

Year, Emerging Pathogen	Impact on the population	WHO’s response	Countries affected/ implications
2003, SARS [18]	8422 cases, 916 deaths in 32 countries over 6 months	Response coordinated by WHO and Global Outbreak Alert and Response Network (GOARN) made up of 115 national health services, academic institutions, technical institutions, and individuals.	WHO estimates that the case fatality ratio of SARS ranges from 0 to 50% with an overall estimate of case fatality of 14–15% China, Hong Kong, Canada, Singapore, Taiwan, and Vietnam were mainly affected.
2009, H1N1 influenza [19]	526,060 cases 6770 deaths 206 countries have eported	Declared Public Health Emergency of International Concern (PHEIC) on April 25, 2009 Declared of Global pandemic on July 1, 2009 Declared as post pandemic on August 10, 2010	East Asia, South East Asia and 21 African nations.
2014, Ebola [20]	28,652 suspected cases, 15,261 laboratory confirmed cases, 11,325 deaths 10 countries affected	Declared PHEIC on August 8, 2014	Guinea, Sierra Leone, Liberia, Mali, Nigeria Epidemic costed a total of $4.3 billion USD, loss of human resources including health care staff, issues of food security, decrease in cross-border trade.
2015, Zika [21]	86 countries have reported evidence of Zika	Declared PHEIC on February 8, 2016	African Region, Region of the Americas, South-East Asia Region, and Western Pacific Region have autochthonous mosquito-borne transmission.
2019, Covid-19 [1]	184,976 cases, 7529 deaths in 159 countries	Declared PHEIC on January 30, 2020 Declared as Global Pandemic on March 11, 2020	China, Italy, Iran, Spain, UK, Korea, France, Germany, USA, Netherlands are affected in large numbers. Other Asian and South American countries have also been affected.

### Disruption of Women’s SRHRs in LMICs and experiences from Ebola, Zika and Covid-19

The findings of the Guttmacher–Lancet Commission on SRHR show that high-income countries have largely instituted policies that favour holistic implementation of SRHR programmes and services, whereas most LMICs are battling with the reality of prioritising SRHR or are simply offering lip service to it. Sadly, most of these LMICs countries are in Africa and southeast Asia [4]. In addition, when there is an epidemic in these LMICs that have poor resources, there is a double burden on their health systems as existing shortages of health human resources already place an extreme strain on capacity to serve patients, especially for non-emergency care.

Moreover, given that gender inequality exists in all continents, including in Africa, Asia and Latin America [5], and that it has a significant impact on women’s lives, including their health, championing SRHR requires the integration and involvement of men in advancing the SRHR agenda. Championing gender equality and including men in the process is pivotal to successful implementation of SRHR services.

As witnessed during the Ebola Virus Disease (EVD) outbreak, the outbreak created additional delays in the care for women experiencing pregnancy complications, thus leading to adverse outcomes, especially in relation to spontaneous abortions and hemorrhage. In addition, financial and structural barriers to care were significantly affected. The Ebola response architecture resulted in a “five delay” model. This led to an overall confusion around how to apply the EVD case definition. For example, “unexplained bleeding” and “spontaneous abortion” also became indications for the immediate need to transfer the patient to the Ebola Treatment Centers (ETC). As such, many of the obstetric complications were classified as meeting the EVD case definition, leading some women to be more reluctant to present to a public health facility due to fear of being transferred to an ETC. These incidents are well described in the Three Delay Model: Delay in seeking care in poor resource setting because of fear of taking public transportation; Delay in reaching care because of availability of transportation and Delay in receiving care due to lack of pharmaceutical products. These same fears may have also been shared by survivors of sexual violence who may have been more reluctant to come to the clinic for post-rape care for fear of being labeled a suspect case, and, as a result of bleeding, transferred to an ETC. This seems to have resulted in many women and men agreeing to avoid pregnancy during the EVD outbreak and therefore continuing to seek family planning services. Unfortunately, these people face an additional hurdle since contraception (beyond condoms) is not systematically offered to EVD survivors or providers at ETCs [6].

The UNFPA’s report [7] from Sierra Leone highlights the challenges faced by women, particularly those in labour, due to their concern that fearful health providers lack adequate protection and training in infection prevention control and Ebola case management. Moreover, since socio-cultural norms dictate that women tend to sick family members, nurse children, and work as traditional healers and healthcare assistants [8], women are at a high risk of infection. Evidence from previous outbreaks reported by various authors [9, 10] found higher rates of Ebola infection in women than men, largely due to socio-cultural practices, including the role of women as caregivers and their involvement in burial practices. Additionally, women’s increased interaction with the health system may put them at greater risk of infection given the systemic weaknesses discussed earlier. Another UNICEF survey report also indicated that women were avoiding seeking health care, for fear of contracting the disease in health facilities, and routine evidence points to reduced uptake of RMNCAH care since the declaration of the Ebola outbreak [11].

In a recent analysis of Covid-19 pandemic, the Guttmacher Institute penned a report highlighting possible shortages in medications such as contraceptives, antiretrovirals for HIV/AIDS and antibiotics to treat STIs due to disruptions in the supply chains (e.g. the shutdown of several drug manufacturing plants in China due to Covid-19, thus causing delays in the production of generic medicines in India) [12].

### Achieving the UHC and health related SDGs in the context of Women’s health

The 2030 Agenda for Sustainable Development explicitly mentions sexual and reproductive health, with Sustainable Development Goal target 3.7 stating “[b] y 2030, ensure universal access to sexual and reproductive health-care services, including for family planning, information and education, and the integration of reproductive health into national strategies and programmes” [13]. The WHO and UNFPA have a common objective to achieve this goal and the best way to do so is through the achievement of Universal Health Coverage (UHC), which would allow everyone to obtain the health services they need, when and where they need them, without facing financial hardship. Doing so will require the government to reach all vulnerable populations with a full range of quality services that are based on their needs, such as: contraceptive services; maternal and newborn care; prevention and control of sexually transmitted infections (STIs), including HIV; comprehensive sexuality education; safe abortion care, including post-abortion care; prevention, detection, and counselling for gender-based violence; prevention and treatment of infertility and cervical cancer; and counselling and care for sexual health and wellbeing [14]. However, during the global health emergencies, there is a total reversal of priorities and, as a result, the availability, accessibility and affordability of SRHR services may become challenging especially in resource-poor settings. All these global epidemics have been weakening the health care systems and increasing the barriers to access reproductive health services in the LMICs, especially by impacting the economic, social and personal decision-making of women. As highlighted by Ahmed et al. (2020), policymakers, providers and advocates must be aware of the broad links between global outbreak response and SRHR in order to prepare mitigation strategies [12]. In this time of global pandemic of Covid-19 and the new demand placed on the system to cope with the resulting new demands, Action Canada for Sexual Health and Rights has emphasized in its statement that there are concerns regarding increased wait times to access SRHR services, difficulties in accessing SRHR medications (including contraceptives, hormone therapy and HIV treatment and increased health risks), and increased health risks experienced by pregnant and immune-compromised people. These crisis situations expose the social, economic and health inequalities and emphasize the need for building an equitable world [15]. The report from African Development confirms the impossibility of building resilience to Ebola and future infectious disease shocks in households and communities without also addressing systemic gender inequality [16], national development strategies for EVD response (or any EID), and gender-sensitive recovery that addresses the associated negative impacts on women and girls [17].

## Conclusions

There is a great need for promoting advocacy and raising awareness of harmful traditional and cultural practices that leave women vulnerable. The national development plans and strategies must be pro-women. It is also very critical to provide effective health facilities and health delivery systems in order to achieve the UN SDG target 3.7 and universal access to SRHR services. The LMICs have been experiencing constant and nuanced threats to SRHRs and given their predisposition to the EIDs, the lessons learned will continue to be relevant for all future outbreaks. Hence the LMICs need to be more proactive by improving coordination at all levels and implementing evidence-based strategies to provide sustainable solutions and thus reduce the demand-service gaps, especially for women’s reproductive health and rights.

## Data Availability

Not applicable.

## References

[1] WHO. Coronavirus disease 2019 (Covid-19): Situation report. Available at https://experience.arcgis.com/experience/685d0ace521648f8a5beeeee1b9125cd [Accessed 15 Mar 2020].

[2] World Health Organization, Regional Office for South-East Asia (2014). A brief guide to emerging infectious diseases and zoonoses. WHO Regional Office for South-East Asia.

[3] Johns Hopkins Medicine. What are Emerging Infectious Diseases? Available at https://www.hopkinsmedicine.org/health/conditions-and-diseases/emerging-infectious-diseases. [Accessed 16 Mar 2020].

[4] Starrs A, Ezeh AC, Barker G (2018). Accelerate progress—sexual and reproductive health and rights for all: report of the Guttmacher–lancet commission. Lancet..

[5] UNDP. Africa human development report 2016: accelerating gender equality and women's empowerment in Africa. Available at http://www.undp.org/content/undp/en/home/librarypage/hdr/2016-africa-human-development-report.html. [Accessed 17 Mar 2020].

[6] McKay G, Black B, Mbambu Kahamba S, Wheeler E, Mearns S, Janvrin A (2019). Not All That Bleeds Is Ebola: How has the DRC Ebola outbreak impacted Sexual and Reproductive Health in North-Kivu?.

[7] UNFPA (2015). Rapid Assessment of Ebola Impact on Reproductive Health Services and Service Seeking Behaviour in Sierra Leone.

[8] Kamara J (2014). We can no longer ignore Ebola’s wider impact – particularly on women.

[9] Hewlett BS, Amola RP (2003). Cultural contexts of Ebola in northern Uganda. Emerg Infect Dis.

[10] Jamieson DJ, Uyeki TM, Callaghan WM, Meaney-Delman D, Rasmussen SA (2014). What obstetrician gynecologists should know about Ebola: a perspective from the centers for disease control and prevention. Obstet Gynecol.

[11] UNICEF. Sierra Leone Health Facilities Survey 2014: Revised Preliminary Findings. In PowerPoint presentation, November 8, 2014, Sierra Leone. Available at https://reliefweb.int/sites/reliefweb.int/files/resources/UNFPA%20study%20_synthesis_March%2025_final.pdf. [Accessed 17 Mar 2020].

[12] Ahmed Z, Sonfield A. The Covid-19 outbreak: potential fallout for sexual and reproductive health and rights. Guttmacher Institute, 2020. Available at https://www.guttmacher.org/article/2020/03/covid-19-outbreak-potential-fallout-sexual-and-reproductive-health-and-rights. [Accessed 16 Mar 2020].

[13] UN Transforming our world: the 2030 Agenda for Sustainable Development. A/RES/70/1. United Nations, New York 2015..

[14] Ghebreyesus TA, Kanem N (2018). Defining sexual and reproductive health and rights for all. Lancet.

[15] Statement on Covid-19. Action Canada for Sexual Health and Rights. Available at https://www.actioncanadashr.org/news/2020-03-13-statement-covid-19. [Accessed 16 Mar 2020].

[16] African Development Bank. Women’s Resilience: Integrating Gender in the Response to Ebola. Available at https://www.afdb.org/fileadmin/uploads/afdb/Documents/Generic-Documents/AfDB_Women_s_Resilience__Integrating_Gender_in_the_Response_to_Ebola.pdf. [Accessed 18 Mar 2020].

[17] UNDP. Africa Policy Note. Confronting the gender impact of Ebola virus disease in Guinea, Liberia and Sierra Leone. Retrieved from https://www.africa.undp.org/content/rba/en/home/presscenter/articles/2015/02/27/ebola-no-lasting-recovery-without-a-special-focus-on-women-says-undp/. Accessed 17 Mar 2020.

[18] WHO. Summary table of SARS cases by country, 1 November 2002–7 August 2003. Available at https://www.who.int/csr/sars/country/country2003_08_15.pdf?ua=1. [Accessed 17 Mar 2020].

[19] WHO. Pandemic (H1N1) 2009- update 75, weekly update, 20 November 2009. Available at https://www.who.int/csr/don/2009_11_20a/en/. [Accessed 17 Mar 2020].

[20] Chattu VK (2017). Politics of Ebola and the critical role of global health diplomacy for the CARICOM. J Family Med Prim Care.

[21] Sikka V, Chattu VK, Popli RK, Galwankar SC, Kelkar D, Sawicki SG, Stawicki SP, Papadimos TJ (2016). The emergence of Zika virus as a global health security threat: a review and a consensus statement of the INDUSEM Joint Working Group (JWG). J Glob Infect Dis.

